# Improving rare disease classification using imperfect knowledge graph

**DOI:** 10.1186/s12911-019-0938-1

**Published:** 2019-12-05

**Authors:** Xuedong Li, Yue Wang, Dongwu Wang, Walter Yuan, Dezhong Peng, Qiaozhu Mei

**Affiliations:** 1 College of Computer Science, Sichuan University, Chengdu, China; 2 School of Information and Library Science, University of North Carolina at Chapel Hill, Chapel Hill, NC United States; 3 MobLab Inc., Pasadena, CA United States; 4 School of Information, University of Michigan, Ann Arbor, MI United States

**Keywords:** Rare disease diagnosis, Knowledge graph, Machine learning, Text classification, Extremely imbalanced data

## Abstract

**Background:**

Accurately recognizing rare diseases based on symptom description is an important task in patient triage, early risk stratification, and target therapies. However, due to the very nature of rare diseases, the lack of historical data poses a great challenge to machine learning-based approaches. On the other hand, medical knowledge in automatically constructed knowledge graphs (KGs) has the potential to compensate the lack of labeled training examples. This work aims to develop a rare disease classification algorithm that makes effective use of a knowledge graph, even when the graph is imperfect.

**Method:**

We develop a text classification algorithm that represents a document as a combination of a “bag of words” and a “bag of knowledge terms,” where a “knowledge term” is a term shared between the document and the subgraph of KG relevant to the disease classification task. We use two Chinese disease diagnosis corpora to evaluate the algorithm. The first one, HaoDaiFu, contains 51,374 chief complaints categorized into 805 diseases. The second data set, ChinaRe, contains 86,663 patient descriptions categorized into 44 disease categories.

**Results:**

On the two evaluation data sets, the proposed algorithm delivers robust performance and outperforms a wide range of baselines, including resampling, deep learning, and feature selection approaches. Both classification-based metric (macro-averaged *F*_1_ score) and ranking-based metric (mean reciprocal rank) are used in evaluation.

**Conclusion:**

Medical knowledge in large-scale knowledge graphs can be effectively leveraged to improve rare diseases classification models, even when the knowledge graph is incomplete.

## Background

A disease is defined as *rare* if it affects fewer than 1 in 2000 people in Europe [1], or it affects fewer than 200,000 people in the United States (1 in 1500 people) [2]. China has recently released its first national list of rare diseases [3]. Across the globe, hundreds of millions of people could be affected by one of about 6000 known rare diseases [4].

Accurate diagnosis of rare diseases is an important task in patient triage, risk stratification, and targeted therapies. Rare disease symptoms often appear unfamiliar and atypical to a clinician, as the cases are too rare to encounter [5]. This brings significant challenge for clinicians to diagnose rare diseases timely, and calls for machine-assisted diagnosis methods.

Rare disease diagnosis is challenging to machine learning approaches as well. Machine learning algorithms often require a significant number of training examples to achieve a good generalization performance. However, by the very nature of rare diseases, the number of relevant clinical records is bounded by the size of population. To compensate the lack of training data for rare disease diagnosis, we need to make use of domain knowledge. Recent efforts in information extraction and knowledge engineering communities have created large-scale knowledge graphs [6–8], in which a large number of entities and relations are extracted from unstructured and semi-structured data, verified manually or semi-automatically, and then organized into a massive graph. Although many of these knowledge graphs are freely available as web-based services, most of them have limited coverage and accuracy. They are often built without considering downstream machine learning tasks, therefore imperfect from a task point of view. In this paper, we are interested in leveraging such knowledge resources in machine-assisted rare disease diagnosis.

We present a simple and effective statistical learning method that improves rare disease classification using an imperfect knowledge graph. We define a rare disease in its statistical sense, i.e. a disease that affects a small percentage (in this paper, less than 0.1%) of the population in a large disease diagnosis corpus. The proposed method is based on the intuition that if a rare disease has a corresponding entity in the knowledge graph, then we can use this piece of knowledge to guide the classifier on “where to focus” when examining a clinical document. This proves to be an effective strategy in classifying rare diseases when the training documents are too few for the algorithm to learn informative features. On two disease classification corpora, the proposed method demonstrates robust improvements over strong baseline methods on rare diseases diagnosis.

### Prior work

**Machine-assisted rare disease diagnosis**. Machine-assisted diagnosis approaches have attracted various lines of research recently [5]. Svenstrup et al. developed a search system that, given symptoms as a search query, returns probable rare disease diagnosis [9]. MacLeod et al. applied gradient boosted decision tree classifiers on behavioral survey data to identify potential rare diseases. Shen et al. proposed a neighborhood-based collaborative filtering algorithm, where patients with similar phenotypes receive similar diagnosis [10]. Their follow-up work further incorporated phenotype-disease associations in biomedical literature [11] and biomedical ontology [12] to improve disease recommendation results. In the current work, we approach rare disease diagnosis in a multiclass classification formulation, which has been shown to deliver state-of-the-art performance in Web-scale applications like ranking and recommendation [13, 14].

**Imbalanced data classification**. From a machine learning perspective, rare diseases in a patient population can be viewed as rare classes in a data set, which is a typical example of imbalanced data set. We can therefore consider imbalanced learning techniques in rare disease classification [15]. Typical imbalanced learning techniques include resampling, cost-sensitive learning, and rare class data synthesis [16]. However, typical machine learning research deals with class imbalance ratios between 1:4 and 1:100, and few recent works tackle imbalance ratio as extreme as 1:1,000 or lower [17, 18]. In this study, we only consider resampling as one of the potential methods, as its performance closely resembles that of cost-sensitive learning, and synthesizing text documents from rare classes is itself a challenging task.

**Feature engineering**. When training documents are too few to provide high-quality features, various feature engineering techniques can help enhance data representation. Feature selection methods can be used to identify informative features for the classification task and discard irrelevant features to alleviate overfitting, especially for high-dimensional data such as text [19]. Instead of reducing features, feature generation aims to add features using external knowledge [20]. The technique first identifies a set of knowledge concepts related to a given document, and then “appends” informative words in these concepts to the document. In between the above two strategies are feature labeling and highlighting, which originated from interactive machine learning literature [21–23]. These methods use domain knowledge to identify a subset of existing informative features, then incorporate them as certain type of informative prior in subsequent classifier training process. In this study, we evaluate various feature engineering methods for integrating domain knowledge into disease classification algorithm.

## Methods

### Data Description and Problem Formulation

We start by describing the two corpora and the knowledge graph used in our study, followed by our definition of rare diseases, all of which lead to our problem formulation.

**Corpora: HaoDaiFu and ChinaRe**. We use two Chinese patient diagnosis corpora. The first corpus, HaoDaiFu, contains 51,374 patient records categorized into 805 diseases. Each document contains the symptom description submitted by a patient to Haodf.com, the largest Chinese online platform that connects patients to doctors. These patients have been previously diagnosed by a clinician, and now come to the platform for further consultation. The second corpus, ChinaRe, contains 86,663 patient records categorized into 44 disease categories. Each document contains the symptom description of a patient written by an insurance professional in ChinaRe, which is one of the largest reinsurance groups in China. The diagnoses were determined by a clinician and sent to the insurance company. Table 1 summarizes basic statistics of the two corpora. Jieba package was used for Chinese word segmentation [24].

**Table 1:** Corpora statistics

	HaoDaiFu	ChinaRe
# of documents	51,374	86,663
# of classes (diseases)	805	44
Vocabulary size	59,879	41,087
Average # of words/doc	26.7	29.7
Average # of knowledge terms/doc	10.8	4.0

A “knowledge terms” is a term appearing in medical knowledge graph (see “Acquiring knowledge features from KG entities” section)

**Fig. 1:**
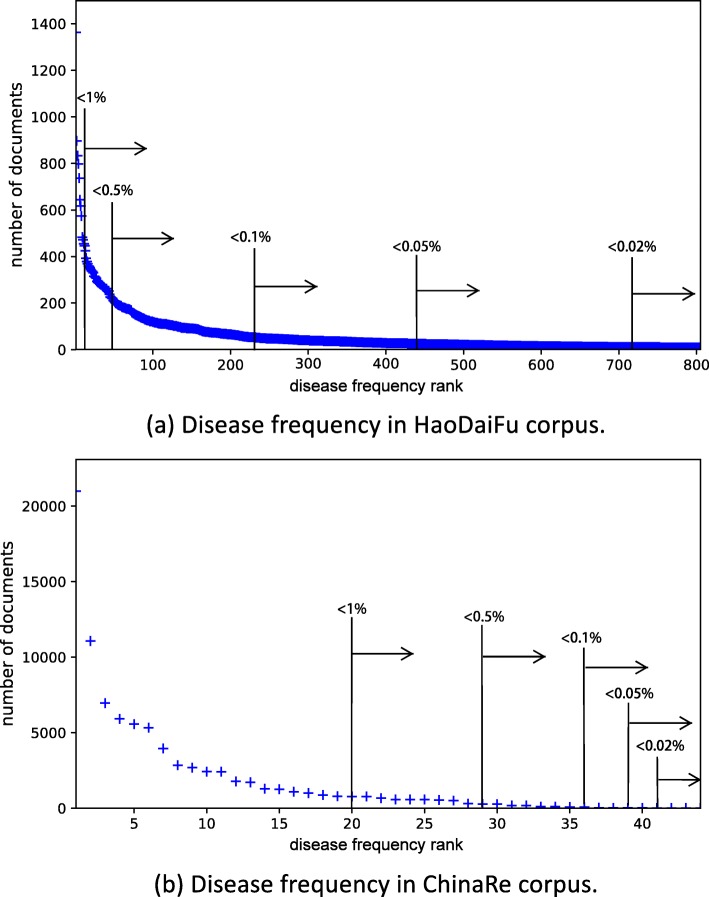
Zipf’s plots of disease frequency in the two corpora. The *x*-axis is the disease frequency rank; the *y*-axis is the disease frequency (number of documents in the disease category). Common diseases appear on the left; rare diseases correspond to the long tail on the right. We annotate cutoff ranks above which the diseases are rarer than the specified percentage

Figure 1 shows disease distributions of the two corpora. We see that both distributions are highly skewed: a few diseases account for thousands of people, while many diseases affect a small percentage of the population.

**Fig. 2:**
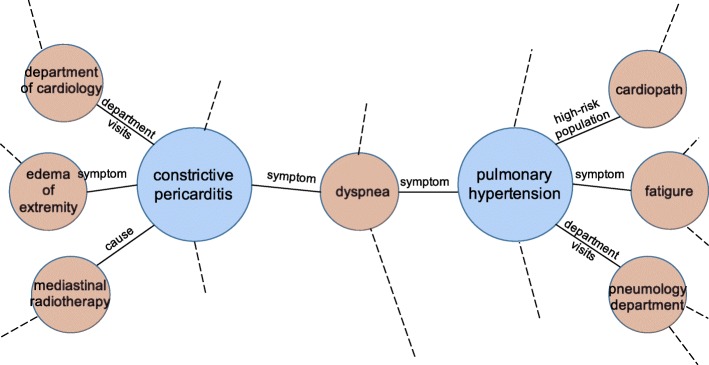
An illustrative example of two disease entities and some of their attributes in a knowledge graph

**Knowledge graph: CN-DBpedia**. A knowledge graph (KG), also known as an ontology, is a collection of entities and relations between entities. An entity has a set of attributes, some of which may itself be an entity. Figure 2 illustrates a small part of a medical KG.

In our study, it would be ideal to have a well-curated medical KG. Unfortunately there is no equivalent of English medical KG like the Unified Medical Language System (UMLS) in Chinese. As it is challenging to guarantee accurate translation of an English KG to Chinese using machine translation, only a small fraction of UMLS concepts has Chinese translation. We leave this direction for future work. We therefore resort to a general Chinese knowledge graph, CN-DBpedia [25]. It aggregates knowledge from various resources and constructed in a similar manner as DBpedia. At the time of writing, it contains 16,892,423 entities and 223,137,127 relations. We use a web-based platform that provides RESTful API access to CN-DBpedia (Knowledge Works [26]). Given a textual query, the API returns matched entities. This allows us to perform entity linking relatively easily. Since CN-DBpedia is automatically constructed from Chinese equivalents of Wikipedia, it does not have perfect coverage over all medical entities, and the crowd-contributed medical content may be inaccurate or incomplete. Not all diseases in the above two corpora have a corresponding entity in the current CN-DBpedia. We find an entity for 751 out of 805 diseases in HaoDaifu and 37 out of 44 diseases in ChinaRe.

**Rare disease definition**. Since different countries and regions adopt different definitions of rare diseases [1, 2], and new rare diseases continue to be registered [3], there is no commonly accepted definition of rare diseases.

For the purpose of this study, we define a rare disease in its statistical sense: a disease is rare if it affects no more than a small percentage of the patient records in a large diagnosis corpus. We set the percentage to 0.1%, or 1/1,000, which is slightly higher than the 1/1,500 – 1/2,000 threshold used in the United States and Europe, since both corpora are biased samples of the entire population, *i.e.*, missing the healthy sub-population. This definition allows us to develop and evaluate algorithms on a wide variety of statistically rare diseases observed in empirical data. In HaoDaiFu, 571 diseases have a percentage lower than 0.1% of all the records. In ChinaRe, 10 diseases have a percentage lower than 0.1% of all the records.

**Problem formulation**. Our goal is to build text classification algorithms that can automatically assign a disease label given the narrative description of a patient’s symptoms. Besides a set of training documents, we also assume access to an existing knowledge graph that contains an entity for (at least a subset of) the diseases in question. In this paper, we specifically focus on classifying rare diseases, or diseases accounting for no more than 0.1% of records in a corpus.

### Knowledge Graph Enhanced Rare Disease Classification

**Fig. 3:**
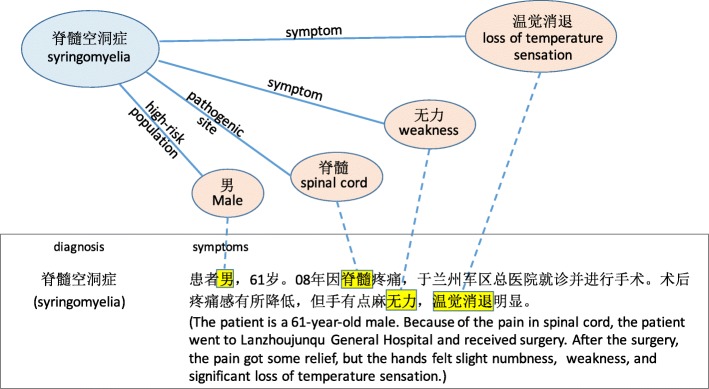
An illustrative example of using knowledge graph to “emphasize” features (words) in a document. This is an ideal case, where the highlighted features are relevant to the diagnosis. In practice, all features that appear in diagnosis-related part of KG will be highlighted

This section describes the proposed method for KG-enhanced rare disease classification. The basic idea is to use external knowledge to “emphasize” existing features in the classifier. To illustrate, let us consider a concrete example in Fig. 3. Suppose we want to detect the rare disease *syringomyelia* in text, but the training documents are extremely few (in the HaoDaifu corpus, 12 out of 41,105, or 1 out of 3425 records). A text classifier essentially aims to identify important words among many irrelevant words that indicate *syringomyelia*. This is a difficult task given the very few training documents and a large vocabulary of words. How can we identify important features, assuming we have access to a KG? A natural strategy is to look up the entity *syringomyelia* in the KG, take the attributes that describe this entity, and “inform” the classifier that words mentioned in the attributes are important features. Figure 3 illustrates this idea.

Below we describe our method in detail. It comprises of three steps: (1) To identify relevant KG entity (or entities) for each disease; (2) To extract important word features from a given KG entity; (3) To incorporate the importance of features into a text classifier.

#### Mapping diseases to KG entities

In this step, we use the KG API to map a disease to the corresponding KG entity. The API performs entity linking and resolves different surface forms (or “mentions”) to the same entity, e.g. mapping “cancer” and “malignancy” to the *cancer* entity. Some disease names may have ambiguous matches. For instance, *insomnia* matches both a health-related entity and a song. To filter out non-medical entities, we further check the category attribute of an entity. We call it the *matched entity* of a disease.

As discussed before, some diseases cannot be mapped to an entity due to the incompleteness of the KG in use. We devised a fall-back strategy to handle these cases. The goal here is to identify not the exact, but the most relevant, entity of a disease. To do so, we evaluate the content overlap between a disease (represented by high inverse document frequency words in all documents of a disease) and an entity (represented by words in its various attributes), and select the entity with the highest content overlap. We call it the *surrogate entity* of a disease.

As a real example, the KG API did not find an entity for *complex congenital heart disease*, so we resort to a surrogate entity *antiarrhythmics* (a drug for heart rhythm disorders) which shares many content words with this disease.

#### Acquiring knowledge features from KG entities

In the following discussion, we use *V* to denote the native word features found in all training documents, where Chinese stop words are removed.

If a disease has a matched entity, we use words in its attributes and related entity names to form disease features. Accumulating over all diseases, we obtain a set of words *K*_1_. *K*_1_ has overlap with *V* but may also contain words not in *V*.

If a disease has a surrogate entity, we do not extract features as above because unlike a matched entity, the attributes of a surrogate entity are highly likely to be irrelevant to the associated disease. We only extract words that appear at least once in any training document of the disease and appear in 0.01% of KG entities (to ensure specificity – similar to the idea of inverse document frequency). This gives us a set of words *K*_2_. By construction, *K*_2_⊂*V*.

In the above example, the surrogate entity *antiarrhythmics* and the training documents of *complex congenital heart disease* share words such as “heart”, “atrium”, “arrhythmia”, “severe”, and “syndrome”. These *antiarrhythmics*-related words are used to detect the presence of *complex congenital heart disease*. They can be helpful but may also introduce errors, depending on their relevance to the actual disease.

We call the union set *K*=*K*_1_∪*K*_2_*knowledge features*, or *knowledge terms*.

#### Integrating knowledge features into text classifier

**Choice of text classifier**. We employed one-vs-rest support vector machine (SVM) classifier with linear kernel, sparse bag-of-words (BOW) feature representation. We found that dense representation methods such as long short-term memory (LSTM) networks perform comparably with sparse SVM on frequent diseases but much worse on rare diseases, with or without pretrained word vectors. In later experiments, we still include the LSTM for comparison.

**Feature vector construction**. Given BOW feature set *V* and knowledge feature set *K*, we construct the feature vector for a document *d* as follows (*d* is viewed as a set of words):

1. Construct a |*V*|-dimensional count vector for BOW features, then apply TF-IDF (term frequency-inverse document frequency [27]) transformation and *L*_2_ length normalization;
2. Construct a |*V*∩*K*|-dimensional count vector for knowledge features in *d*∩*K*, then apply TF-IDF transformation and *L*_2_ length normalization;
3. Concatenate the above two vectors to represent the document.

The first step constructs a feature vector for the original document. The second step constructs a feature vector for words in the document that are mentioned in KG (*d*∩*K*). Concatenating feature vectors is also called *early fusion* in multimodal learning, where different vector segments correspond to different modalities of the same data [28].

If a document contains a word *w*∈*V*∩*K*, then it will appear twice in the feature vector: one as a BOW feature, the other as a knowledge feature. Note that the two feature values will not be identical, since the two vectors will have different *L*_2_ lengths before normalization. Such a word will receive a larger feature value in the second vector, since the “KG-mentioned part” (*d*∩*K*) is shorter than the original document (*d*). Table 1 shows that each document in HaoDaiFu has 26.7 words on average, in which 10.8 words are knowledge features. The ratio is lower in ChinaRe (4.0/29.7). Therefore, the second feature vector can be understood as *emphasizing knowledge features in a document*.

### Experimental Evaluation

In this section, we evaluate the effectiveness of proposed method and a suite of baseline settings on the rare diseases in the two corpora.

**Train-test split**. To reduce the variance of results due to a random train-test split, we average the results of 10 runs. In each run, we randomly split the corpus into 80% for training and 20% for test. To avoid the case where some classes do not appear in training or test set, the random split is applied on a per-class basis.

#### Compared methods

Except for LSTM, all compared methods use one-vs-rest linear SVM classifier, sparse feature representation. We performed grid search for the hyperparameter *C* over {0.001, 0.01, 0.1, 1, 10, 100} on a validation set, and found that *C*=1 consistently delivered the best performance to the baseline method **BOW** (described below). We set *C*=1 in all SVM classifiers.

Methods that do not make use of knowledge features:

1. **BOW**: only use BOW feature vector in “Integrating knowledge features into text classifier” section.
2. **LSTM**: the long short-term memory neural networks, hidden state size =256, randomly initialized word vectors (slightly higher performance on rare classes than pretrained word vectors).
3. **UpSample**: upsample the rare disease documents in the training set, so that each disease has equal number of documents. This is a standard method for imbalanced classification.
4. ***χ***^2^: use |*V*∩*K*_1_| features selected by the *χ*^2^ criterion. We want to compare the efficacy of features selected by external knowledge (KG) vs. standard feature selection method (*χ*^2^).
5. **BOW**+ ***χ***^2^: concatenate the BOW and *χ*^2^ feature vectors in the same manner as in “Integrating knowledge features into text classifier” section.

Methods that make use of knowledge features:

1. **KG**_1_: only use *V*∩*K*_1_ as features;
2. **KG**_12_: only use (*V*∩*K*_1_)∪*K*_2_ as features;
3. **BOW+KG**$_{1}^{\text {early-fusion}}$: concatenate BOW and KG_1_ feature vectors as in “Integrating knowledge features into text classifier” section;
4. **BOW+KG**$_{12}^{\text {early-fusion}}$: concatenate BOW and KG_12_ feature vectors as in “Integrating knowledge features into text classifier” section.

Other variants that also make use of both BOW features and KG_1_ features:

1. **BOW+KG**$_{1}^{\text {late-fusion}}$: the late fusion strategy (as opposed to early fusion/concatenating features in multimodal learning [28]): we combine two SVM predictions: one trained on BOW vectors, the other trained on KG_1_ vectors. To combine the predictions for each document, we rank the predicted labels from most to least probable, and combine the two predicted lists using Borda’s rank aggregation method [29].
2. **BOW+KG**$_{1}^{\text {pseudo-count}}$: the pseudo count strategy [21]: concatenating KG features to BOW is equivalent to increasing the corresponding BOW feature values, which in turn is equivalent to increasing corresponding word counts. For each word in a given document that also appears in KG_1_, we add *k* pseudo word counts to the BOW feature vector. We tuned *k*=1,10,100 and set *k*=1 as it gives the best performance.
3. **BOW+KG**$_{1}^{\text {pseudo-doc}}$: the pseudo document strategy: we view the mention of a knowledge feature in a training document as annotating the rationale of the label. We then use the rationale learning strategy to generate pseudo training documents [30].

#### Evaluation metrics

To evaluate the effect of different methods at different rarity levels, we bin the diseases by their percentage in a corpus. Three bins are below 0.1% (our definition of *rare* diseases):

- (0 – 0.02%]: no more than 1/5,000;
- (0.02% – 0.05%]: 1/5,000 to 1/2000;
- (0.05% – 0.1%]: 1/2,000 to 1/1,000.

For a comprehensive comparison, we also include two bins between 0.1% and 1%:

- (0.1% – 0.5%]: 1/1,000 to 1/200;
- (0.5% – 1%]: 1/200 to 1/100.

## Results

Machine-assisted diagnosis can be viewed both as a classification task (to assign a disease label to a document) and a ranking task (to sort disease labels by their relevance to a document). To evaluate the classification performance, we use macro-averaged *F*_1_ score [31] as it balances precision and recall and is not biased by majority classes. To evaluate the retrieval performance, we use mean reciprocal rank (MRR) [32] since in both corpora, each document has only one associated disease. We report macro-averaged *F*_1_ and MRR on the test data of each bin.

In Tables 2 and 3, we report macro-averaged *F*_1_ and MRR results in each bin across different methods. Statistical significance of these results against the BOW baseline is assessed by randomization test [33]. We set the type I error control at *α*=0.05.

**Table 2:** Rare disease classification performance on HaoDaiFu corpus

*Percentage Bins*	(0, 0.02%]		(0.02%, 0.05%]		(0.05%, 0.1%]		(0.1%, 0.5%]		(0.5%, 1%]	
	89 diseases		277 diseases		205 diseases		194 diseases		32 diseases	
	*F*_1_	MRR	*F*_1_	MRR	*F*_1_	MRR	*F*_1_	MRR	*F*_1_	MRR
BOW	34.10	45.86	40.80	49.91	49.48	58.81	**53.23**	**62.80**	**62.23**	**75.31**
LSTM	0.00	0.41	0.01	1.07	0.38	5.91	12.29	27.23	40.07	53.04
UpSample	35.17 ^∗^	47.10 ^∗^	40.69	50.43 ^∗^	47.63	57.63	49.85	59.75	58.6	68.95
*χ*^2^	34.04	46.75 ^∗^	40.81 ^∗^	50.66 ^∗^	49.15	58.53	51.74	61.38	61.55	74.05
BOW+ *χ*^2^	34.56	47.25 ^∗^	42.41	**51.84** ^∗^	50.03 ^∗^	**59.33** ^∗^	53.15	62.34	62.10	73.97
KG_1_	33.66	44.98	38.25	47.45	45.17	53.97	48.07	57.55	59.21	71.29
KG_12_	33.51	44.92	39.08	48.07	45.23	54.55	48.66	58.00	59.2	71.43
BOW+KG$^{\text {pseudo-doc}}_{1}$	31.91	42.81	37.51	46.08	44.08	53.22	47.01	56.94	55.91	69.47
BOW+KG$^{\text {pseudo-count}}_{1}$	34.87 ^∗^	46.14 ^∗^	41.74 ^∗^	50.14 ^∗^	49.31	57.94	52.56	61.59	61.65	74.19
BOW+KG$^{\text {late-fusion}}_{1}$	33.33	45.42	38.41	48.68	47.15	56.39	51.13	60.18	61.42	73.30
BOW+KG$^{\text {early-fusion}}_{1}$	36.87	**48.36** ^∗^	**43.11** ^∗^	51.79 ^∗^	**50.06** ^∗^	58.99	52.86	61.90	61.90	73.57
BOW+KG$^{\text {early-fusion}}_{12}$	**36.94** ^∗^	48.22 ^∗^	42.63 ^∗^	51.40 ^∗^	49.66	58.62	52.60	61.51	61.47	73.23

The higher *F*_1_ and MMR, the better. Each column’s highest number is shown in **boldface**, second highest number shown with underline. The left three percentage bins are rare disease bins; the right two bins are for comparison purposes. “ ^∗^” denotes results significantly higher than BOW (randomization test, significance level *α*=0.05)

**Table 3:** Rare disease classification performance on ChinaRe corpus

*Percentage Bins*	(0, 0.02%]		(0.02%, 0.05%]		(0.05%, 0.1%]		(0.1%, 0.5%]		(0.5%, 1%]	
	5 diseases		3 diseases		2 diseases		7 diseases		9 diseases	
	*F*_1_	MRR	*F*_1_	MRR	*F*_1_	MRR	*F*_1_	MRR	*F*_1_	MRR
BOW	91.58	93.36	29.76	53.97	90.49	93.49	88.69	92.64	92.6	95.09
LSTM	0.00	4.03	0.00	4.75	0.00	9.64	22.38	44.68	85.86	93.55
UpSample	88.36	94.81	52.22	66.54	90.11	93.06	89.36	94.27	92.62	95.76
*χ*^2^	91.38	95.83 ^∗^	47.97	65.12	90.40	93.68	91.92	95.41	93.84	**96.45**
BOW+ *χ*^2^	93.37 ^∗^	97.55 ^∗^	42.14 ^∗^	62.80 ^∗^	90.73	93.95	**92.01**	**95.55**	**94.05**	96.43
KG_1_	91.06	97.47 ^∗^	22.63	43.64	48.52	48.11	80.54	86.67	74.32	77.33
KG_12_	92.26 ^∗^	**97.70** ^∗^	31.20	43.91	85.61	91.42	83.71	87.96	80.05	83.18
BOW+KG$^{\text {pseudo-doc}}_{1}$	75.68	82.49	34.86	52.08	83.20	87.84	78.79	85.57	88.34	91.86
BOW+KG$^{\text {pseudo-count}}_{1}$	88.14	91.02	30.04 ^∗^	52.62	89.02	93.61	85.54	88.64	90.8	93.34
BOW+KG$^{\text {late-fusion}}_{1}$	89.01	95.41 ^∗^	29.76	48.8	68.63	70.80	86.18	89.65	86.89	86.21
BOW+KG$^{\text {early-fusion}}_{1}$	92.30 ^∗^	97.66 ^∗^	**54.73** ^∗^	**69.88**	90.27	92.54	91.00	95.05	93.59	95.92
BOW+KG$^{\text {early-fusion}}_{12}$	**93.43** ^∗^	97.13	47.78	62.04	**91.68**	**95.41**	90.70	94.49	93.46	95.70

See the footnote below Table 2 for details

## Discussion

First, we observe that for rare diseases (three bins under 0.1%), the proposed methods BOW+KG$_{1}^{\text {early-fusion}}$ and BOW+KG$_{12}^{\text {early-fusion}}$ deliver robust performance: they are almost always among the top two performers on both corpora. As the disease becomes less rare (two bins above 0.1%), simple BOW baseline and supervised feature selection work better. This is expected as the proposed methods can be viewed as doing feature selection using external knowledge. With more training data in each class, the knowledge inside training data allows us to select higher quality, more task-specific features than external knowledge.

In the disease-to-KG-entity mapping step (“Mapping diseases to KG entities” section), including surrogate entities is sometimes beneficial to rare disease classification, but not always. The performance gain of having higher entity coverage (BOW+KG_12_ compared to BOW+KG_1_) is the most salient when the disease is extremely rare (below 0.02%). This suggests that if we had a more complete KG, the rare disease classification performance could be even better.

The performance of LSTM is extremely low on rare diseases. Indeed, deep learning methods need a large quantity of training data to perform well, which are unavailable for rare classes in the long tail. Using pretrained word vectors did not help, since rare classes have far less training documents than frequent classes to fine-tune the relevant word vectors.

The performance of upsampling is very unstable, which agrees with previous literature [16]. It dramatically improves classification performance in one specific case (ChinaRe, 0.02% – 0.05%). But in most other cases, upsampling does not help or even hurts performance compared to the BOW baseline. Combining upsampling with other methods (e.g. *χ*^2^ or KG_1_) results in even more unstable performance, which we omit. This suggests that resampling is not suitable for extremely imbalanced text classification tasks.

On rare diseases, concatenating the vectors of original BOW features and knowledge features tends to perform better than using either alone, for both *χ*^2^-selected features and KG-selected features. We can understand this phenomenon as a type of regularization: the selected feature segment can be understood as “to put emphasis on these features”. Or equivalently, it can be understood as “to reduce attention (lower the weights) on the rest of the BOW features”. To illustrate this, Table 4 shows examples of learned feature weights that for the rare disease *syringomyelia*. Conceptually, this is related to group-wise regularization: to apply different regularization strengths on two groups of features: *V*∩*K* and *V*∖*K*. The problem with group-wise regularization is that for each disease, we would need a different hyperparameter to balance the strength of regularization on two feature groups. The proposed method does not have this problem.

**Table 4:** Example feature weights of the rare disease *syringomyelia*

Feature	BOW	BOW+KG$_{1}^{\text {early-fusion}}$
Syrinx	1.19	1.34
Temperature sensation	0.52	0.82
Numb	0.76	0.45
Tremble	0.82	0.75

Our method BOW+KG$_{1}^{\text {early-fusion}}$ learned to place larger weights on knowledge features (“syrinx” and “temperature sensation”) and smaller weights on non-knowledge features (“numb” and “tremble”)

Among different ways of using the KG feature information, we found that early fusion performs the best. Combining classification predictions (late fusion) is challenging at the global level, since the combination weights might be different for different diseases. The pseudo-count method has no significant effect, because incrementing the count of an existing term by 1 has diminishing effect after TF-IDF transformation. On the other hand, a large pseudo-count makes the document vector as if containing only selected features. Instead, allocating additional dimensions for these features turns out to be more beneficial. It has been shown that text classification can benefit from having many redundant but not perfectly correlated features [34]. Finally, the pseudo-example method performs poorly because it generates more examples for large classes, making small classes even smaller.

### Implication

One of the biggest challenges in applying machine learning techniques to healthcare is the lack of supervision signals in this domain. Unlike other domains (e.g., image, speech) where the availability of training labels is bounded by the annotation budget, in healthcare it is bounded by the availability of domain experts, and in the case of (rare) diseases, also bounded by the population of patients [35]. How to efficiently transfer domain knowledge into supervision signals for training machine learning models has been a heated debate in both the research community and industry of medical NLP. Under resource constraints, should the effort be spent on labeling additional training examples, or constructing knowledge graphs? Despite many potential advantages of knowledge graphs over unstructured annotations (e.g., precise and compact knowledge representation, extendable, reusable for different tasks [36]), there is always a concern that building a complete and accurate KG can be labor-intensive, if not impossible.

This work shows that a knowledge graph does not have to be perfect (in terms of coverage and accuracy) to be able to deliver desirable benefits for medical NLP tasks. Our use of a general-purpose KG also indicates that practitioners could start with customizing and refining an open domain KG for their tasks instead of building a medical KG from the scratch. Our results should resolve some of the concerns of building knowledge graphs in the practices of medical NLP.

## Conclusion

This paper studied the problem of rare disease classification, where rare diseases are defined by their presence in a large corpus (lower than 0.1%). We developed a text classification algorithm that represents a document as a combination of a “bag of words” and a “bag of knowledge terms”, where a “knowledge term” is a term shared between the document and the subgraph of knowledge graph relevant to the disease classification task. On two Chinese disease classification corpora, the algorithm delivers robust performance gain over feature selection methods on rare diseases.

In future work, we plan to explore a variety of methods for improving document representation. First, instead of “emphasizing” all words that appear in medical-related KG, we can do so more selectively. One way is to identify the most relevant KG entities to a specific document, and only emphasize words in those entities. We can use synonyms and word embedding methods to allow for fuzzy matching between KG entities and a document, to increase the coverage of knowledge features in a document. We can also consider “appending” words in relevant entities to a document, effectively performing feature generation. Finally, when medical experts are interacting with a list of predicted rare diseases or most similar patients, we can explore the opportunity of learning from experts feedback and improve the diagnosis algorithm continuously.

## Data Availability

Not applicable.

## Abbreviations

BOW: Bag-of-words
API: Application programming interface
KG: Knowledge graph
LSTM: Long short-term memory
UMLS: Unified medical language system
